# Effect of Material Properties on the Foaming Behaviors of PP-Based Wood Polymer Composites Prepared with the Application of Spherical Cavity Mixer

**DOI:** 10.3390/polym13183179

**Published:** 2021-09-19

**Authors:** Suwei Wang, Ping Xue, Wenxin Zhang, Gazi Hao, Lei Xiao, Wei Jiang

**Affiliations:** 1 National Special Superfine Powder Engineering Research Center of China, School of Chemistry and Chemical Engineering, Nanjing University of Science and Technology, Nanjing 210094, China; wangsw90@163.com (S.W.); hgznjust1989@163.com (G.H.); 15005161138@163.com (L.X.); superfine_jw@126.com (W.J.); 2 College of Mechanical and Electrical Engineering, Beijing University of Chemical Technology, Beijing 100029, China; 3 Guannan County Electric Power Corporation, Lianyungang 222002, China; br13812326643@163.com

**Keywords:** wood-polymer composites, polypropylene, cavity transfer mixer, molecular configuration, cell morphology

## Abstract

For the low weight and high strength, the microcellular extrusion foaming technology was applied in the preparation of polypropylene (PP)-based wood polymer composites, and the spherical cavity mixer was used to construct an experimental platform for the uniform dispersion of wood flour (WF). The effects of PP molecular configuration on the composite properties and cell morphology of samples were also investigated. The experimental results indicated that the application of a spherical cavity mixer with a cavity radius of 5 mm could effectively improve the mixing quality and avoid the agglomeration of WF. In addition, compared with the branched molecule, the linear molecule not only increased the melting temperature by about 10 °C, but also endowed composites with a higher complex viscosity at a shear rate lower than 100 s^−1^, which contributed to the cell morphology of more microporous samples.

## 1. Introduction

In recent decades, the rapid development of society not only consumed a large amount of timber and polymers, but also created innumerable waste plastics and wood, which cause serious environmental pollution and a huge shortage of forest resources [1,2,3]. Therefore, to realize the sustainable development of society, more attention was paid to the reutilization of wastes. Meanwhile, composites with waste plastics and wood flour (WF), when used as raw materials, have great advantage in terms of their environmental protection, high chemical resistance and good processing characteristics. Therefore, they are deemed to be a kind of high-value-added, green, environmental protection material [4,5].

Accordingly, wood–polymer composites (WPCs) have gradually replaced the application of timber, and show broad development prospects in interior decoration, architectural decoration, packaging and transportation, automobile manufacturing and other fields [6]. In addition, not only the raw materials, but also the products made of WPCs, can be recycled and reused [7,8]. The emergence and development of WPCs are conducive to the recycling of waste resources, the alleviation of resource shortage and the protection of forest resources.

Previous studies have indicated that WPCs possess the characteristics of both wood and plastic: they are water-resistant, reusable, and have a high chemical resistance and good processing characteristics [9]. Nevertheless, the much higher density of WPCs has seriously limited their popularization; they can be more than two times as dense as natural wood [7,10]. Besides this, the excessive addition of WF could reduce the toughness, impact strength and bending strength of WPCs, which limits the high-performance application of WPCs.

However, the introduction of a uniform microporous structure was found to be effective in improving the ductility while decreasing the density of composites, which is mainly attributed to the fact that the cracks inside materials were effectively suppressed by the micropores, leading to the increased growth energy [11,12]. As the homogenous system of melt/gas was the prerequisite for the microcellular structure, the uneven dispersion of WF particles often results in a larger cell diameter and lower cell density than neat polymers [13,14,15,16]. 

As a result, the cavity transfer mixer (CTM) was designed and applied to construe the foaming experimental platform with a single screw extruder. Meanwhile, PPs with different molecular configurations were chosen as the matrix, and the thermal and rheological properties of composites were characterized and analyzed. Moreover, the effect of WF content and molecular structure on the morphology of composites was also investigated, aiming to explore the evolution law of bubbles and promote the microporosization of composites.

## 2. Experimental Platform

### 2.1. Structural Parameters of CTM

**Figure 1:**
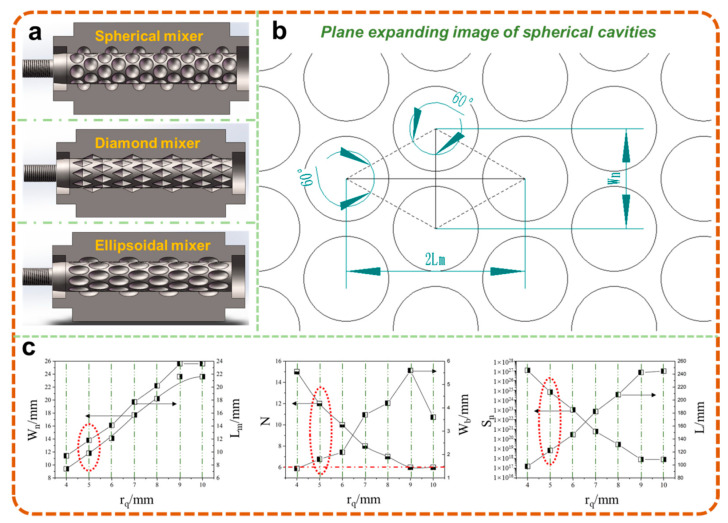
Mixers with different cavity shapes (**a**) and plane expanding image of spherical cavities (**b**), and parameters of spherical cavity mixer with different cavity sizes (**c**).

According to the previous work, the addition of WF endowed the composites with a higher apparent viscosity, which not only deformed the static mixer, but also resulted in more difficulty in the dispersion of the strong agglomeration between the WF particles. Therefore, the cavity transfer dynamic mixer with a strong shear dispersion ability was applied in the extrusion of composites with high viscosity, as shown in Figure 1a [17,18]. Meanwhile, considering the processing difficulty and cost, the mixer with spherical cavities was used as a screw accessory to achieve the uniform distribution of components; the cavity plan of a rotor was shown in Figure 1b [17,18,19].

The dispersive mixing ability of a spherical cavity transfer mixer was mainly decided by the number of circumferential and axial cavities, which could be described as the number of striations [20,21]

(1) $$S_{n}=N^{2M-1}$$

where *S_n_* is the theoretical number of mixer striations, *N* is the number of circumferential cavities, and *M* is the number of axial cavities. The distance between adjacent cavities and other structural mixer parameters could also be denoted by *N* and *M*, which were calculated as follows:

(2) $$W_{n}=\frac{2\pi R_{z}}{N}$$

(3) $$L_{m}=\frac{\sqrt{3}}{2}W_{n}=\frac{\sqrt{3}\pi R_{z}}{N}$$

(4) $$w_{b}=\frac{2\pi R_{z}}{N}-2r_{q}$$

(5) $$L=2r_{q}+\frac{\sqrt{3}\pi R_{z}}{N}(M-1)$$

where *W_n_* is the circumferential distance between adjacent cavities, *R_z_* is the radius of rotor, *L_m_* is the axial distance between adjacent cavities, *w_b_* is the minimum wall thickness between adjacent cavities, *r_q_* is the cavity radius, L is the effective rotor length. Considering the poor interfacial compatibility between WF and PP and the strong agglomeration among WF particles, the number of circumferential cavities M was set as 12, and the detailed parameters were listed in Figure 1c. 

As shown in Figure 1c, the increased *r_q_* not only led to an increased distance between adjacent cavities, it also resulted in a decreased number of axial cavities M and a decrease in the theoretical number of striations *S_n_*, which indicated that the mixer had an impaired mixing quality. Meanwhile, in order to avoid the problem of cavity interference during drilling, the minimum wall thickness was kept higher than 1.5 mm. As a result, the structural parameters of the spherical cavity mixer were determined with the *r_q_* of 5 mm, which was then fixed at the end of the screw in threaded connection.

### 2.2. Effect of CTM Application 

**Figure 2:**
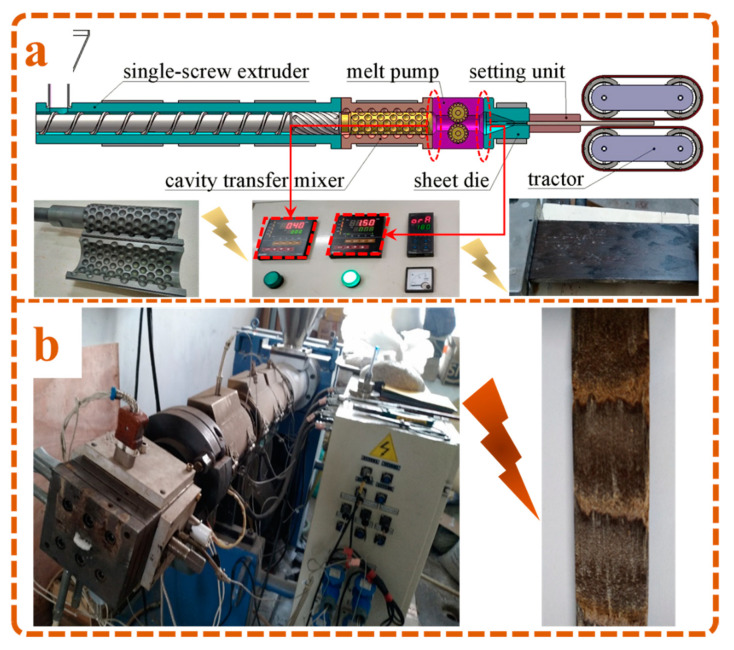
Experimental platform for the microcellular foaming of PP/WF composites: (**a**) Extruder with the application of CTM and the prepared samples; (**b**) Extruder without the application of CTM and the prepared samples.

Based on the designed spherical cavity mixer shown above, the experimental platforms with/without CTM were shown in Figure 2a,b, respectively. Compared with the smooth surface of the sample in Figure 2a, the exposure of agglomerated WF in Figure 2b indicated the poor apparent quality of the sample prepared by the platform without CTM. Thus, the mixing ability of the designed CTM could effectively improve the dispersion uniformity of WF and meet the microcellular foaming requirements of PP-based WPCs.

However, the decreased results of the pressure sensor shown in Figure 2a also revealed the pressure loss exerted by spherical CTM on melt during extrusion, which was mainly attributed to the energy consumption of the mixing used for screw rotation and melt conveying. Therefore, a melt pump was connected in series with the equipment by screws, which could significantly improve the die pressure and meet the pressure drop demand during melt foaming. According to the above descriptions, the experimental platform used for the microcellular foaming of WPCs was constructed and shown in Figure 2a.

## 3. Experimental

### 3.1. Materials

The PP-based WPCs mainly consisted of resin matrix, WF and other fillers. In this study, two types of PP with different molecular structures, PP1 (B8101) and PP2(B1002W), were applied as the resin matrix, both purchased from Sinopec Yanshan Petrochemical Company (Beijing, China). The poplar WF (HC wooden Co., Quzhou, China) with a size of 150 μm, talc (Haicheng Talc Powder Manufacturer, Haicheng, China) with a size of 23 μm and MAH-g-PP (CA100, Arkema Co., Ltd., Paris, France) were purchased and used as the main fillers. In addition, the bubbles among the samples were achieved by the blowing agent Azodicarbonamide (AC), which was purchased from Selon Industrial Co (Leping, China). with a gas production of 220 mL/g and decomposition temperature of 160 °C.

### 3.2. Preparation of Samples

**Figure 3:**
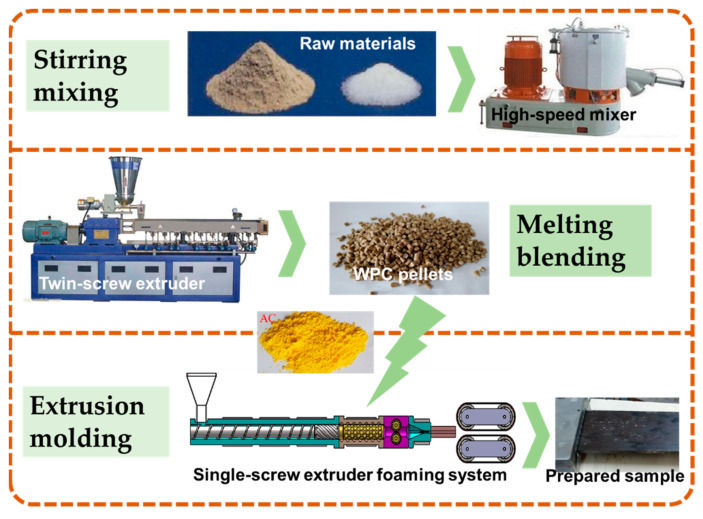
Preparation process of PP-based WPCs.

As shown in Figure 3, the preparation process of PP-based WPCs can be divided into stirring mixing, melting blending and extrusion molding. First, the resin matrix, WF and other fillers were mixed in turn by a high-speed mixer (SHR-25A, Yongli Mechanical Co., Zhangjiagang, China) for 30 min, and then melt blended by a co-rotating twin-screw extruder with a length diameter ratio of 40 (Φ20 mm, Kesun Rubber & Plastic Machinery Co., Kunshan, China). Before mixing, the WF was dried in an oven at 80 °C for 24 h to remove the moisture, and the mass ratio of each component was also listed in Table 1.

Second, the prepared WPC pellets were mixed with the foaming agent AC, and then extruded into the continuous plate with 80 mm × 8 mm rectangular section by the constructed experimental platform. Finally last, the plates were cut into specimens of certain shapes for subsequent testing.

### 3.3. Characterization

#### 3.3.1. Melt Flow Rate (MFR)

The tester RL-Z1B1, provided by Shanghai S.R.D. Scientific Instrument Co., Ltd. (Shanghai, China) was applied to measure the melt flow rate of materials, while the temperature and the load were set as 230 °C and 5 kg, respectively. The mass flow rate was calculated as follows:

(6) $$MFR=600\times \frac{m}{t}$$

where *MFR* is the mass flow rate of melt (g/10 min), *m* is the mass of splines (g), and *t* is the sample time interval (s). The *MFR* value of each sample was averaged after five repetitions.

#### 3.3.2. Rheological Property

The rheological properties of the materials were characterized by complex viscosity (η), shear storage modulus G′, loss modulus G′′ and loss tangent (tanδ), which were conducted on a parallel-plate rheometer HAAKE MARS III, provided by Thermo Fisher Scientific Inc. (Shanghai China).The rheological tests were performed between parallel plates with the diameter of 25 mm and gap of 1 mm at 180 °C, which were also conducted in dynamic mode and nitrogen atmosphere. The frequency sweeps between 0.01 and 100 rad/s were carried out at strains within the linear viscoelastic range and the scan amplitude was 1% during testing.

#### 3.3.3. Thermal Property

The thermal properties of composites were characterized by the crystallization behavior, which was measured by differential scanning calorimeter (DSC). 

In the DSC test, the samples were heated up to 210 °C at a rate of 10 °C /min and held for 1 min to eliminate heat history, then cooled down to 25 °C at the same rate, before being analyzed by DSC Q2000, provided by TA Instruments Co. (New Castle, Germany) in nitrogen atmosphere. The crystallinity *X_c_* of PP was calculated as follows:

(7) $$X_{c}=\frac{\Delta H_{f}}{\Delta H_{f}^{o}\times \omega }$$

where ∆*H*_f_ is the heat of fusion generated during melting or crystallization, ∆*H*_f_^o^ is the theoretical heat of fusion of PP with 100% crystallinity (209 J/g [22]), and *ω* is the mass fraction of PP.

#### 3.3.4. Micromorphology

The samples’ micromorphology was observed by a stereomicroscope SZM-45T2 provided by Sunny Optical Technology Co., Ltd (Yuyao, China). The fracture surfaces of the samples were observed by scanning electron microscope (SEM) (S-4700, Japan Hitachi Company, Tokyo, Japan) after tensile tests, and the fracture surfaces were coated with a thin gold layer before observation.

#### 3.3.5. Mechanical Property

The mechanical properties of the samples were characterized by tensile strength and flexural strength, which were both tested with a load of 5 kN and speed of 10 mm/min by the universal tester KXWW, provided by Taiding Testing Machine Manufacturing Co., Ltd (Chengde, China). The value of each sample was averaged after five repetitions.

## 4. Results and Discussion

### 4.1. Effect of Molecular Structure on the Properties of Composites

#### 4.1.1. Thermal Property

**Figure 4:**
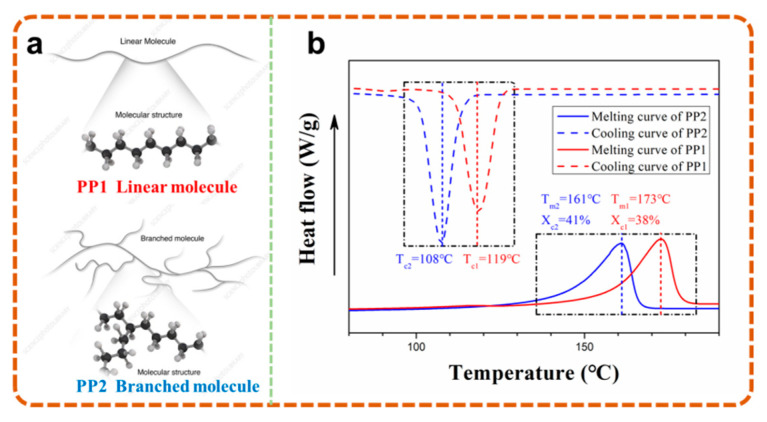
Molecular configuration (**a**) and thermal properties (**b**) of PP1 and PP2.

The foaming temperature was mainly determined by the thermal properties of the composites, which were greatly affected by the molecular structure of PP [23,24]. As shown in Figure 4a, compared with the propylene-ethylene random copolymer PP1 with linear molecule, the branched molecule endowed PP2 with a wider molecular weight distribution and relaxation time distribution. The wider molecular weight distribution of PP2 promoted the growth of trigonal crystal [25], while the introduction of an ethylene segment destroyed the continuity of the propylene segment and transformed the PP1 macromolecular chain into triclinic crystal with a high number of polypropylene short chains. When affected by the crystal, both the melting temperature and crystallization temperature of PP1, listed in Figure 4b, were about 10 °C higher than in PP2.

**Figure 5:**
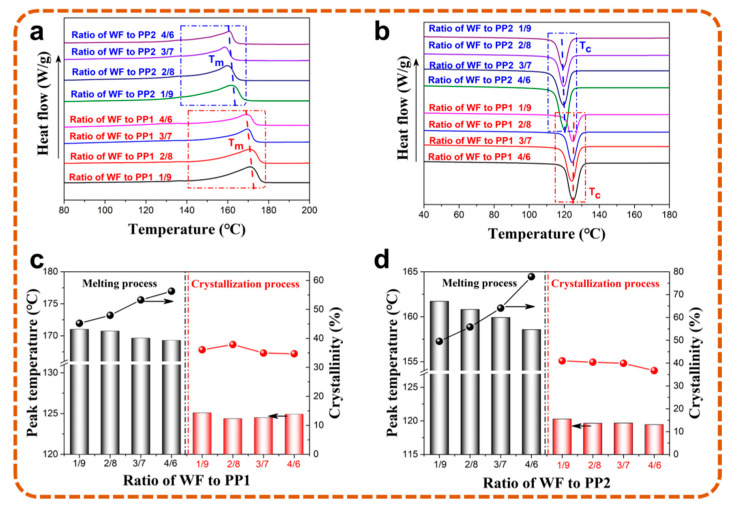
DSC curves (**a**,**b**) and thermal properties (**c**,**d**) of PP1 and PP2 based composites with different WF content.

The melting and crystallization behaviors of composites with different WF contents were both characterized by differential scanning calorimetry (DSC), and the thermal properties are also shown in Figure 5. According to the listed data, the peak values of DSC curves with the matrix of PP1 were much higher than PP2-based composites at the same WF content. The addition of WF caused heterogeneous nucleation and promoted the perfection of crystal morphology, which led to the increased crystallinity and the decreased peak temperatures [26]. During extrusion, the mixed WF could reduce the energy used for crystallization, which converted more amorphous chains into crystals and resulted in the narrower peak. Thus, the crystallinity of PP1-based composites increased by 24.5% as the ratio of WF to PP increased from 1/9 to 4/6, while it was 57.2% for PP2-based composites.

#### 4.1.2. Melt Flow Rate

**Figure 6:**
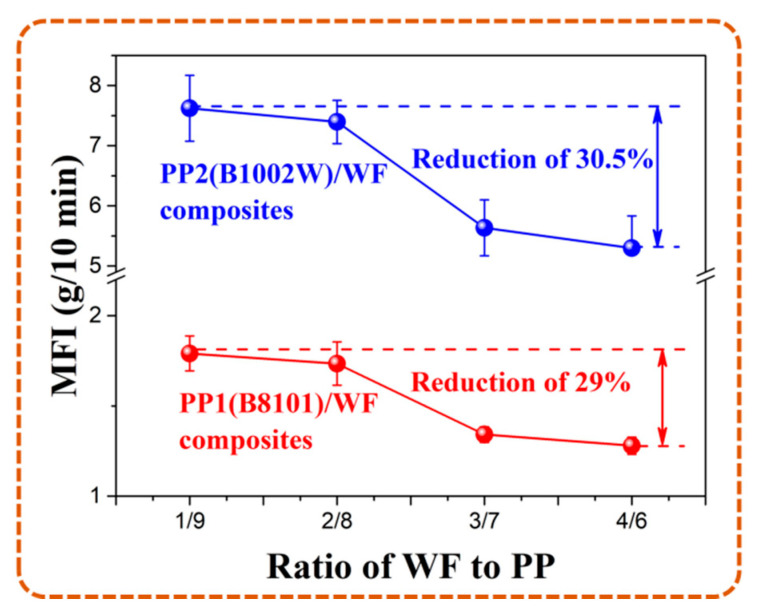
MFR of PP1 and PP2 based WPCs with different WF content.

To describe the flowability of composites, MFRs of melt with different WF contents and resin matrixes were measured and shown in Figure 6. The data listed below indicate that the addition of WF could increase the melt viscosity of composites, and a great decrease in MFR occurred at the ratio of 3/7. On the one hand, WF particles dispersed in the matrix acted as physical interlocks for the formation of ester bonds with PP macromolecules, which hindered the movement of molecular chains [27,28]. The hindrance was also gradually enhanced with the increase in WF content. On the other hand, excessive WF content could enhance the probability of contact between particles, which also results in intense internal friction and a decrease in flowability.

#### 4.1.3. Rheological Property

**Figure 7:**
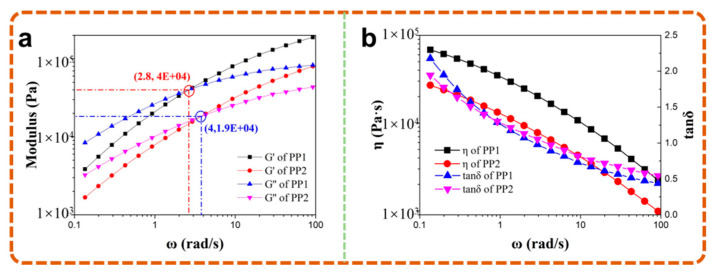
Modulus (**a**), complex viscosity and loss tangent (**b**) of materials.

On the basis of the composites’ MFR, the frequency dependences of molten-state oscillatory shear moduli were also investigated and are shown in Figure 7. It can be seen from Figure 7 that the complex viscosity of PP1 was much higher than that of PP2 in the low-frequency region. However, with the increase in angular frequency, the difference in viscosity between PP2 and PP1 got smaller. This is mainly because, in the low-frequency region, the shear rate of the melt was small, and the molecular chain had enough time to relax, which contributed to restoration of the conformation of the molecular chain that was changed by the external force. Therefore, the viscosity in the low-frequency region was mainly affected by the density of the entangled nodes. The more nodes were entangled, the greater the intermolecular force and the higher the resin viscosity. With the increase in shear rate, PP molecular chains were oriented along the direction of melt flow under the action of shear stress, and there was not enough time to relax, which resulted in a decreased number of entangled nodes between the molecular chains and the phenomenon of shear thinning.

Meanwhile, the angular frequency at the intersection of storage modulus (G’) and loss modulus (G”) curves could reflect the relaxation time and weight-averaged molecular weight of PP [29,30]. The smaller angular frequency at the intersection indicated a longer relaxation time and larger average molecular weight of PP molecular chains, while the smaller value of G’ at the intersection represented the wider molecular weight distribution of PP. Additionally, the abscissa and ordinate values of PP1 and PP2 were calculated and listed in Table 2.

**Figure 8:**
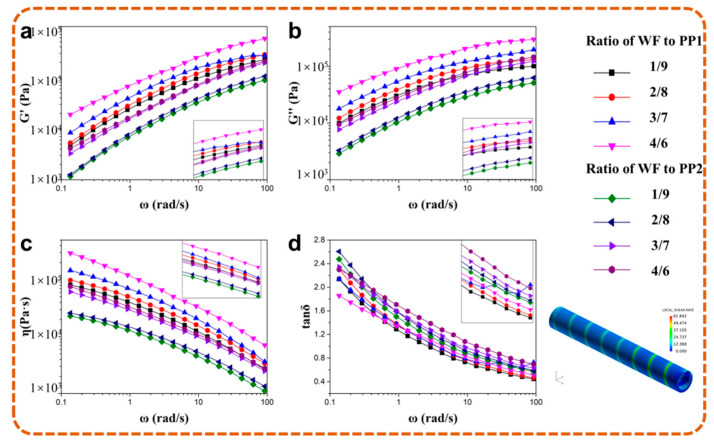
Storage modulus G′ (**a**), loss modulus G′′ (**b**), complex viscosity η (**c**) and loss tangent tanδ (**d**) of PP_1_ and PP_2_ based composites with different WF content.

According to the rheological characteristic parameters, the larger molecular weight of PP1 decreased the difficulty in the formation of entanglements, which caused the much higher viscosity of PP1 compared to PP2. As shown in Figure 8, PP1-based wood-polymer composites showed a higher storage modulus, loss modulus and complex viscosity than PP2-based composites, which were affected by the rheological properties of the matrix. 

However, compared with linear macromolecules, the branched macromolecules had a great advantage in reducing the effect of shear action on the viscosity of resin. Therefore, with the increase in shear rate, the decrease in of PP1 viscosity was larger than that of PP2, which was more obvious in the rheological curves of the composites. In addition, both PP1 and PP2 were dominated by a viscous loss in the low-frequency region, and the loss factor was more sensitive to the movement of PP molecular chains than the movement of viscosity. The higher changing amplitude of PP1 indicated that it had a poorer stability than PP2.

#### 4.1.4. Mechanical Property

**Figure 9:**
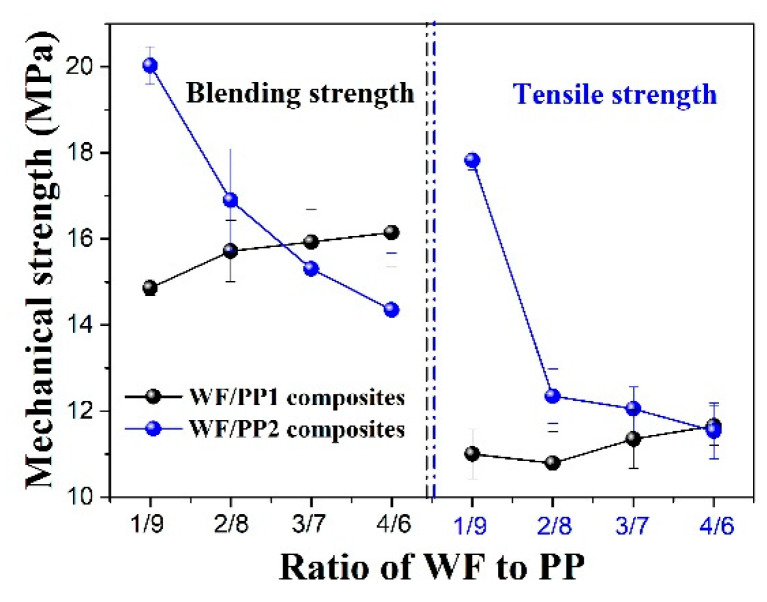
Blending strength and tensile strength of composites with different resin matrix.

The mechanical strengths of composites with a different matrix were shown in Figure 9. According to the listed data, the addition of WF caused a great decrease in the mechanical strength of composites with the PP2 matrix, while a small increase in PP1. This can mainly be attributed to the fact that, compared with the branched molecule of PP2, the linear molecule of PP1 enhanced the physical entanglement of WF, which also led to the higher blending and tensile strength of PP1-based composites at a higher WF content. 

### 4.2. Effect of Molecular Structure on the Microstructure of Samples

#### 4.2.1. Dispersion State of Fillers

**Figure 10:**
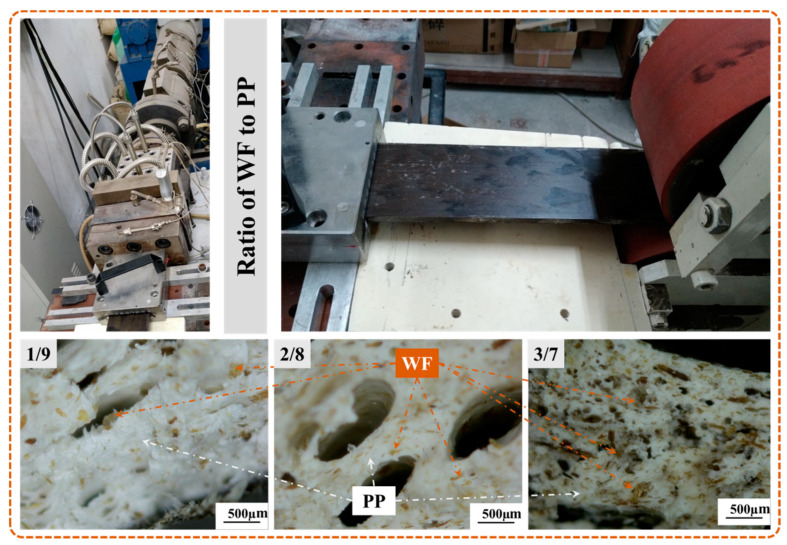
Dispersion state of fillers in the composites with different WF content.

Apart from their apparent quality, the dispersion state of fillers on the samples’ section was observed, by an optical microscope, to characterize the platform’s mixing effect. As shown in Figure 10, the yellowish discrete elements represented WF particles, while the PP matrix was represented by the white continuous phase. The micrographs indicated that the increased WF content could enlarge the distribution density of fillers in the matrix, while no obvious reunion phenomenon existed. In summary, the application of CTM not only improved the apparent quality of the sample, but also achieved the uniform dispersion of the yellowish WF particles, which effectively avoided the problem of bubble coalescence caused by agglomeration. 

#### 4.2.2. Cell Morphology on Axial Section

**Figure 11:**
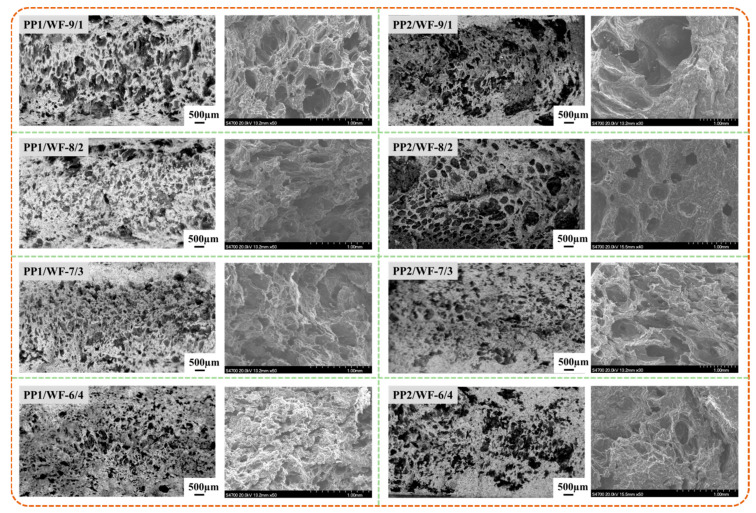
Cell morphology of axial section of samples with different WF contents.

**Figure 12:**
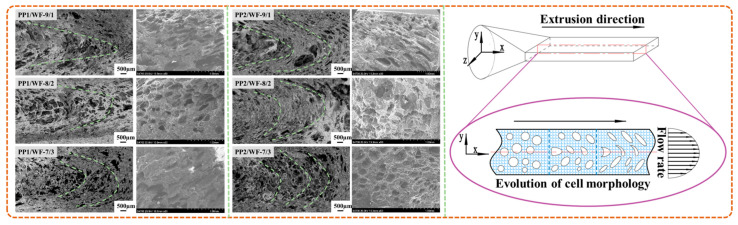
Cell morphology on radial section of samples with different WF content and the evolution process of bubble structure.

The mechanical properties and density of samples were both affected by the morphology of bubbles, and the sample micrographs for axial and radial sections were shown in Figure 11 and Figure 12, respectively. 

According to the micrographs in Figure 11, the addition of WF particles increased the cell density, which acted as the bubble nucleation point. Meanwhile, the increased melt viscosity of composites mixed with WF also enhanced the bubble growth resistance, which provided the smaller cell size. However, the exorbitant WF content could easily result in the agglomeration of particles and the escape of gas used for bubble growth from the gaps between particles. As a result, the bubble size and density of samples achieved minimum and maximum values at the ratio of WF to PP 3/7.

Moreover, the simulation result shown in Figure 8 indicated that the shear rate was kept lower than 100 s^−1^ during extrusion. Thus, the higher complex viscosity of PP1-based WPCs was achieved, and samples with more uniform cell morphology were prepared. 

#### 4.2.3. Cell morphology on Radial Section

Compared with the bubbles on the axial section, the cell morphology on the radial section was not very regular, which was mainly attributed to the velocity difference existent in the mold channel. M Favelukis et al [31,32,33] found that the bubble core grown in the shear flow field was gradually stretched into a long, narrow ellipsoid along the direction of melt flow, and its stretching degree was greatly affected by melt viscosity, bubble interfacial tension, shear rate and bubble radius.

Contrary to batch foaming and compression molding foaming, bubbles foamed in the flow field with large radial velocity gradient moved in the radial direction of the channel. According to the evolution process of the bubble structure on the radial section (perpendicular to Z axis) shown in Figure 12, the spherical bubbles were not only gradually stretched into a flat ellipsoid shape due to the different melt flow rates along the Y direction, but also arranged at a certain angle with the center line of the flow channel under the action of shear flow field in the die. 

## 5. Conclusions

To realize the microporosization of PP-based WPCs, the effects of spherical CTM and molecular configuration on the cell morphology of samples were investigated. The conclusions from this research are summarized and listed as follows:

(1) Compared with static mixer, CTM with a strong shear dispersion ability was more suitable for the dispersive mixing of materials with high viscosity, which was also improved by the increased radius and axial number of cavities. Meanwhile, the experimental results also demonstrated that the application of spherical CTM with the radius of 5 mm could effectively prevent the exposure of agglomerated WF on the sample surface and realize the uniform dispersion of fillers.
(2) Affected by the molecular configuration, the linear molecular endowed PP1 with the triclinic crystals, which were about 10 °C higher than the trigonal crystals of PP2 with branched molecule. The smaller angular frequency of PP1 at the intersection of G’ and G” curves indicated the longer relaxation time and higher average molecular weight, which decreased the difficulty in the formation of entanglements and caused the much higher viscosity.
(3) The addition of WF and linear molecular endowed PP1-based composites with higher viscosity at a shear rate lower than 100 s^−1^, which contributed to the smaller size and larger density of the bubbles. Moreover, the bubbles foamed in the shear flow field were gradually stretched into a flat ellipsoid shape and arranged along the flow direction for the radial velocity gradient.

## Figures and Tables

**Table 1:** The formulation of PP/POE/wood–flour composites.

Sample	Weight (%)					
	PP1	PP2	WF	MAH-g-PP	Talc	AC
1	90	/	10	3.5	10	1
2	80	/	20	3.5	10	1
3	70	/	30	3.5	10	1
4	60	/	40	3.5	10	1
5	/	90	10	3.5	10	1
6	/	80	20	3.5	10	1
7	/	70	30	3.5	10	1
8	/	60	40	3.5	10	1

**Table 2:** Angular frequency (ω) and storage modulus (G’) at the intersection of modulus curves.

Sample	ω (rad/s)	G’ (Pa)
PP1	4.28	19,729.37
PP2	2.92	42,404.58
